# Characterization of Partially Purified Bacteriocins Produced by *Enterococcus faecium* Strains Isolated from Soybean Paste Active Against *Listeria* spp. and Vancomycin-Resistant Enterococci

**DOI:** 10.3390/microorganisms9051085

**Published:** 2021-05-18

**Authors:** Joanna Ivy Irorita Fugaban, Jorge Enrique Vazquez Bucheli, Wilhelm Heinrich Holzapfel, Svetoslav Dimitrov Todorov

**Affiliations:** ProBacLab, Department of Advanced Convergence, Handong Global University, Pohang 37554, Gyeongbuk, Korea; jifugaban@up.edu.ph (J.I.I.F.); jorge_jbv@hotmail.com (J.E.V.B.); wilhelm@woodapple.net (W.H.H.)

**Keywords:** bacteriocin, fermented food, *Enterococcus faecium*, *Listeria monocytogenes*, vancomycin-resistant enterococcus, enterocin

## Abstract

Three out of one hundred eighty putative LAB isolates from Korean traditional fermented soybean paste were identified to be unique and bacteriocinogenic strains. Based on phenotypic and 16S rRNA sequencing analysis, selected strains were identified as *Enterococcus faecium* ST651ea, *E. faecium* ST7119ea and *E. faecium* ST7319ea. The bacteriocinogenic properties of the studied strains were evaluated against *Listeria monocytogenes* ATCC15313, *Listeria innocua* ATCC33090 and vancomycin-resistant *E. faecium* VRE19 of clinical origin. The strains *E. faecium* ST651ea, ST7119ea and ST7319ea expressed bacteriocins with an activity of 12,800 AU/mL, 25,600 AU/mL and 25,600 AU/mL, respectively, recorded against *L. monocytogenes* ATCC15131. According to the PCR-based screening of bacteriocin-related genes, which was further confirmed through amplicon sequencing, showed that strain *E. faecium* ST651ea carries *ent*B and *ent*P genes, whereas both *E. faecium* ST7119ea and ST7319ea strains harbor *ent*A and *ent*B genes. The molecular size of expressed bacteriocins was estimated by tricine-SDS-PAGE showing an approximative protein size of 4.5 kDa. The assessment of the spectrum of activity of bacteriocins ST651ea, ST7119ea and ST7319ea showed strong activity against most of clinical VRE isolates, majority of other *Enterococcus* spp. and *Listeria* spp. Bacteriocins ST651ea, ST7119ea and ST7319ea were partially purified by combination of 60% ammonium sulfate precipitation and hydrophobic chromatography on the SepPakC_18_ column. Challenge test with semi-purified (60% 2-propanol fraction) bacteriocins resulted in a significant reduction of viable cells for all test organisms. Thus, indicating that all the bacteriocins evaluated can be used as potential biocontrol in food and feed industries as well as an alternative treatment for VRE-related infections in both veterinary and clinical settings.

## 1. Introduction

Even though lactic acid bacteria (LAB) are generally known to be fastidious microorganisms, *Enterococcus* spp., a LAB, has been associated with a wider ecological distribution relative to the other members of the LAB due to their ability to survive in a vast range of pH and temperature and their ability to metabolize varieties of carbohydrates. Their applications as starter cultures and probiotics for some fermented food and feed products [1,2,3], and their emerging significant role in the clinical and veterinary sectors as commensals and opportunistic pathogens- particularly in nosocomial infections such as endocarditis, bacteremia and other related infections [4,5,6,7] have highlighted their importance, may it be a boon or a bane, in the food manufacturing industries, veterinary and clinical settings.

Some of the beneficial metabolites and by-products produced by the members of the enterococci group and other LAB play a significant role in bio-preservation including organic acids, hydrogen peroxide, carbon dioxide, diacetyl and proteinaceous antimicrobials or bacteriocins [8]. However, the conquest for highly stable and mechanistically archetypal antimicrobial active molecules has allowed the shift in the focus for the continuous discovery, study and elucidation of bacteriocins and their mechanisms as compared to the other antimicrobial metabolites produced by various LAB.

Bacteriocins are biologically active peptides that act as inhibitory substances typically against phylogenetically related microorganisms to the producer strains but are inherently resistant to their own. These active peptides are known as primary antimicrobial metabolites in contrast with microorganism-derived antibiotics [9,10,11]. The majority of the known bacteriocins produced by enterococci are classified under the Class I and Class II bacteriocins [12], and few heat-labile bacteriocins including enterolysin A and bacteriocin 41 [13,14,15]. Some of the bacteriocins produced by *Enterococcus* spp. have been found to play a significant role in the food industry as naturally occurring bio-preservatives against food-borne pathogens including *L. monocytogenes*, *L. innocua*, *Bacillus cereus*, *Bacillus* spp., *Staphylococcus aureus*, *Streptococcus mitis* [16,17,18,19,20,21,22,23,24] and even clinically significant drug-resistant strains such as methicillin-resistant *S. aureus* (MRSA) [25] and vancomycin-resistant enterococci (VRE) [23,26]. This possibility of using bacteriocins as a treatment for drug-resistant strains can be advantageous in combating their continuous emergence, particularly in the veterinary and clinical setting, due to misuse of antibiotics. Furthermore, the decline in the discovery of efficient antibiotics is relatively lagging compared to the emergence of “super-bugs”.

Fermented food products, associated with various beneficial metabolites and health benefits, have been long consumed as one of the staple foods for centuries due to their long shelf-life. Aside from this, artisanally produced traditional fermented food products are known as possible sources of naturally occurring beneficial strains present originally from the raw materials used in the food preparation. *Doenjang*, one of the many Korean traditional fermented food products made from soybean, can also be potentially harboring multiple strains of LAB that can be investigated for their various functional properties and bacteriocin production.

Thereby, this study focuses on the evaluation, partial characterization and applications of *Enterococcus faecium* strains and their bacteriocins as a potential biocontrol agent against food-borne pathogen *L. monocytogenes* and its indicator organism *L. innocua*, along with vancomycin-resistant *Enterococcus*, a clinically significant emerging pathogen.

## 2. Materials and Methods

### 2.1. Isolation of Putative LAB Strains and Screening for Bacteriocin Production

Samples of artisanal Korean traditional fermented soy paste (*doenjang*) obtained from local markets in the Pohang region, Korea were randomly and aseptically collected and were transported in cool containers at 8–10 °C for microbiological processing. From these collected samples, 10 g of each were transferred in a dilution bottle containing 90 mL of sterile saline solution (0.85% NaCl, *m*/*v*). Samples were serially diluted (ten-fold) prior to spread-plating on de Man, Rogosa and Sharpe (MRS, Difco Laboratories Inc., Franklin Lakes, NJ, USA) supplemented with 1.5% agar (Difco). Plates were incubated for 24–48 h at 37 °C. Isolates of interest were screened by catalase reaction and gas production from glucose fermentation in parallel with purity evaluation using the standard microbiological assessment including streaking in MRS supplemented with 1.5% agar, macroscopic and microscopic observation of colony morphology and microscopic observation of cell morphology through wet-mount and Gram staining reaction according to the phenotypic characterization recommendations from Bergey’s Manual of Systematic Bacteriology of Archaea and Bacteria [27]. Obtained pure cultures were preserved in the presence of 20% glycerol and stored at −80 °C.

For the screening of bacteriocin-producing strains, cell-free supernatant (CFS) from cultures of interest were collected by centrifugation (8000× *g*, 10 min) from 18 h-old aerobically cultured isolates in MRS broth at 37 °C. The collected supernatant was adjusted to pH 5.5 to 6.5 using a sterile 1 M NaOH, was heat-treated for 10 min at 80 °C to eliminate hydrogen peroxide and inactivate proteolytic enzymes potentially produced in the CFS and filtered using 0.2 µm syringe filters (Sartorius Ministart Syringe hydrophilic Filter, Göttingen, Germany), according to recommendations of Todorov et al. [28]. The prepared CFS was initially tested for the production of antimicrobial metabolite/s against *L. monocytogenes* ATCC15313 and subsequently tested against *L. innocua* ATCC33090, and vancomycin-resistant *E. faecium* 19 (VRE19), a clinical isolate. The plates for the evaluation activity of bacteriocin production were prepared using BHI (Difco) supplemented with 1% agar (Difco), whereas bacterial test culture incorporated have an approximative cell population of 10^5^ CFU/mL. Ten microliters of each CFS were spotted on the agar surface and were left to dry for 30 min. All plates were incubated at 37 °C for 18–24 h, and visible zones of inhibition (at least 2 mm) were considered positive [29]. Initial screening for the bacteriocinogenic activity of the isolates was conducted in triplicates in at least two independent experiments.

### 2.2. Differentiation and Identification of Bacterial Isolates

Isolates showing positive inhibitions on the screening process for the production of antimicrobial metabolite/s were subjected to DNA profiling using RAPD-PCR with the primers OPL-01 (5′-GGC ATG ACC T-3′) and OPL-11 (5′-ACG ATG AGC C-3′) and rep-PCR (5′-(GTG)_5_-3′) [30]. The bacterial DNA was obtained from 18 h cultures, grown on MRS at 37 °C, using ZR Fungal/Bacterial DNA Kit (Zymo Research, Irvine, CA, USA) as recommended by the manufacturer. DNA concentration and purity were assessed using SPECTROstar Nano nanodrop (BMG LABTECH, Ortenberg, Germany). All PCR-amplification experiments were performed in Veriti 96-well thermal cycler, Applied Biosystems (Thermo Scientific, Waltham, MA, USA). The generated amplicons were separated by gel electrophoresis using 1.0% (*w*/*v*) agarose stained with SYBR^®^ Safe DNA Gel Stain (Thermo Scientific) on running conditions of 100 V for 45 min. Visual observation and analysis of generated DNA profiles were performed using Omega Lum™ G gel documenter (Aplegen, Inc., Arbor, CA, USA). The identified unique profiles were further subjected to 16S rRNA partial sequencing, performed as an external service (SolGent Analysis Service, Daejeon, Korea) and the generated sequences were analyzed using Basic Local Alignment Search Tool (BLAST, National Center for Biotechnology Information, Bethesda, MD, USA) for identification.

### 2.3. Evaluation of Nature, Integrity and Stability of the Bioactive Molecule

The proteinaceous nature of the antibacterial substances was confirmed by treating each collected CFS with selected proteolytic enzymes including Proteinase K, α-chymotrypsin and trypsin (all from Sigma Aldrich, St. Louis, MO, USA); and α-amylase for the hydrolysis of carbohydrate moiety possibly present in the structure bioactive compound. The test for activity was carried out according to the recommendations of Muñoz et al. [29]. The cell-free supernatant was prepared as previously described and was treated with the abovementioned enzymes in a final concentration of 0.1 mg/mL and incubated for 1 h at 37 °C before subjecting to heat treatment (95 °C, 10 min) to halt enzymatic reactions. Evaluation of the inhibitory activity was carried out against *L. monocytogenes* ATCC15313 and *E. faecium* VRE19, and further screened against *L. innocua* ATCC33090, by the spot-plate method; untreated CFS was used as the positive control.

Furthermore, the stability of the inhibitory compound was assessed in the presence of various key chemical substances, used in analytical research and industrial food and feed production processes, including milk, glycerol, Tween 20, Tween 80, sodium chloride, ethylenediamine tetraacetic acid (EDTA) and sodium dodecyl sulfate (SDS) (All from Sigma Aldrich) due to their possible interference in the activity of any employed antimicrobial peptides. Two milliliters of CFS of each bacteriocin-producing strain were treated with the chosen chemicals to a final concentration of 10 mg/mL for 1 h at 37 °C. The activity was tested using the same method as previously described but before testing for the bacteriocin activity, pH was adjusted to 5.5–6.0 as needed.

The stability of the inhibitory substance(s) was/were assessed in varying temperatures and pH, independently. The CFS of bacteriocin-producing strains were prepared as described before and were subjected to the following temperatures: −20, 4, 30, 37, 45, 60, 80, 100 and 121 °C for 2 h and 4 h except for 121 °C which was carried out for 15 min at 15 psi. The activity was evaluated on both *L. monocytogenes* ATCC15313 and *E. faecium* VRE19. The effect of pH changes on the stability of the bacteriocin produced by the strains of interest was assessed by adjusting the CFS to the following pH conditions: 2.0, 4.0, 6.0, 8.0 and 10 using 1 M NaOH and 1 M HCl, accordingly. Adjusted CFS were incubated for 1 h at 37 °C before testing for activity. Untreated CFS was used as positive control and all experiments were performed in at least two independent events.

### 2.4. Bacteriocin Production, Growth Kinetics and Fermentation

Bacteriocin producing strains were inoculated in 250 mL of MRS broth with an inoculum ratio of 2% (*v*/*v*) from an 18 h-old culture and was incubated at 37 °C for 24 h, samples were withdrawn during the designated time interval for the assessment of bacteriocin activity, cell growth and pH changes. Whereas the changes in pH were measured using pH meter (ST3100-B pH bench, Ohaus, Parsippany, NJ, USA), optical density at 600 nm was measured using a spectrophotometer (Smart UV-Vis Spectrophotometer, Optizen, Daejon, Korea), and bacterial cell count was measured using a flow cytometer, accordingly on the time interval set, for 27 h.

For the assessment of bacteriocin activity, a two-fold dilution assay was performed using 100 mM potassium phosphate buffer (pH 6.5) to measure activity against *L. monocytogenes* ATCC15313 grown in BHI soft agar. Initial dilution with no observed inhibition was used for the calculation of the activity expressed in arbitrary units per milliliter according to the recommendations of Todorov et al. [28].

Furthermore, an assessment of the growth dynamics was performed. Cells were obtained from the samples drawn at each specific time interval and were tallied using ZE5 Cell Analyzer Flow Cytometer (Bio-Rad Laboratories, Inc, Hercules, CA, USA) and quantified and analyzed using the Everest software. Cells were obtained through centrifugation (8000× *g*, 20 min, 20 °C) from 1 mL of the bacterial culture drawn at each time point. Pellets obtained were washed twice with 1 mL of 1x sterile phosphate buffer saline (PBS, Lonza Bio Whittaker, Basel, Switzerland).

### 2.5. Effect of Varying Incubation Temperature on Bacteriocin Production of Studied E. *faecium* Strains

All selected bacteriocin producing strains were grown in MRS broth (Difco) at temperatures 37, 39, 40, 41, 43 and 45 °C for 18 h. Bacteriocin production and activity were assessed against *L. monocytogenes* ATCC 15313 using the two-fold titration technique, as previously described, to observe the outcome of variations in incubation temperatures in the bacteriocin production of the strains.

### 2.6. Screening for Associated Bacteriocin Genes

The previously isolated DNA was used for the screening of bacteriocin production-related genes. Primers targeting different bacteriocin key structural genes due to their high prevalence in most bacteriocinogenic *Enterococcus* spp., albeit some are atypically occurring, were used for the preliminary screening (enterocins: *ent*A, *ent*B, *ent*P, *ent*L50B; nisin Q: *nis*; and pediocin PA-1: *ped*) as recommended by Barbosa et al. [31]. The primers used were summarized in Table 1. PCR reactions and analyses of generated amplicons were carried out as described previously. The amplicons from the positive results of the initial screening were purified using QIAquick**^®^** PCR Purification kit (Qiagen, Hilden, Germany) according to the manufacturer’s instructions and subjected to sequencing services (Macrogen, Seoul, Korea) for confirmation and comparative analysis relative to the sequences deposited in GenBank through BLAST (as shown in Table 2).

### 2.7. Bacterial Growth Inhibition (Cell Lysis)

Bacteriocins are characterized by a unique or combination of various modes of action for bacterial growth inhibition that is highly associated with which group they belong to. This characteristic plays an important role in the dominance and survival of bacteriocinogenic microorganisms in particular niches. *L. monocytogenes* ATCC15313, *L. innocua* ATCC33090 and *E. faecium* VRE19 were grown in 100 mL of BHI and MRS broth, for *Listeria* spp. and *E. faecium*, respectively, with inoculum concentration of 2% (*v*/*v*) and initially incubated for 3 h. Filter-sterilized (Sartorius Ministart 0.2 µm hydrophilic filter) CFS from the bacteriocinogenic strains were prepared and added to the culture (10% *v*/*v*) and changes in optical density (OD 600 nm) was measured hourly for additional 7 h. The viable cell count of each test organism on the 10th h was measured by withdrawing 100 µL on each corresponding test organism and was spread plated on the corresponding growth media supplemented with 2% agar and incubated for 24 h at 37 °C.

### 2.8. Assessment of the Range of Activity of Bacteriocins Produced by E. faecium Strains

The scope of activity of bacteriocin produced by the *E. faecium* ST651ea, ST7119ea and ST7319ea was assessed against a bacterial test panel mentioned in Table 3. The test panel has consisted of different LAB which has been studied for their distinctive beneficial applications obtained from HEM culture collection (Holzapfel Effective Microbes Inc., Pohang, Korea) and reference strains from ATCC (American Type Culture Collection, Manassas, VA, USA) and KACC (Korean Agricultural Culture Collection, Suwon, Korea), food-borne pathogens from ATCC and vancomycin-resistant *Enterococcus* strains of clinical origin (Laboratory of Antimicrobials, Handong Global University, Pohang, Korea). Test plates containing individual test organism grown for at least 18 h was seeded in BHI soft agar (1% *w*/*v*) to a final cell count of 10^5^ CFU/mL. Previously prepared CFS, as described elsewhere, of each bacteriocin were spot-plated on the agar surface. Plates were left to dry for 30 min before incubating at 37 °C for 18–24 h. Inhibition zones were observed, wherein zones of at least 2 mm were considered positive [29].

### 2.9. Quantification of Adhered Bacteriocin to the Producer Cells

The quantification of adhered bacteriocin to the producer cells was performed according to Yang et al. [38]. The 18 h-old cultures of producer strains were all adjusted to pH 6.0, subsequently, the cells were harvested (10,000× *g*, 15 min, 4 °C) and washed with sterile 100 mM potassium phosphate buffer (pH 6.5) before re-suspending with 10 mL of 100 mM NaCl (pH 2.0). The cells were incubated at 4 °C with shaking for 1 h. The cell-free supernatant was obtained and neutralized to pH 7.0 with sterile NaOH. Bacteriocin activity was tested as a two-fold titration technique and quantified as previously described [28]. Each set-up was performed in at least two independent experiments.

### 2.10. Adsorption of Bacteriocin to the Test Organisms

The quantification of adsorption of bacteriocins produced by *E. faecium* ST651ea, ST7119ea and ST7319ea was assessed based on the method proposed by Biscola et al. [39] using two sets of test panel consisted of susceptible and non-susceptible sets of test organisms. The susceptible test panel includes *L. monocytogenes* ATCC15313, *L. innocua* ATCC33090 and *E. faecium* VRE19, while the non-susceptible test panel includes *L. plantarum* ATCC14197 and *L. rhamnosus* LGG.

Test organisms were grown in BHI or MRS according to culture collection recommendations for 18 h at 37 °C. Bacterial cells of each test organism were harvested (4000× *g*, 30 min, 4 °C), washed twice with sterile 100 mM potassium phosphate buffer (pH 6.5) and subsequently re-suspended on the same buffer to reach 0.5 reading at OD 600nm. Each test organism was subjected to each bacteriocin with the ratio of 1:1 (bacterial cell suspension to CFS containing bacteriocin) with a total working suspension of 1.6 mL. The working suspensions were incubated at 37 °C for 1 h. Residual bacteriocin activity from the working suspension was all assessed through a two-fold titration method as previously described. Reduction of bacteriocin activity equates to the total adsorbed bacteriocin of the target microorganism, which was quantified as follows:

$$\%\;\mathrm{adsorption}=\left(100-\frac{AU/mL_{t1}}{AU/mL_{t0}}\right)\times 100,$$

wherein AU/mL_t0_ and AU/mL_t1_ are the bacteriocin activity before and after treatment, respectively. Each set-up was conducted in at least two independent experiments.

Using the susceptible test panel, adsorption of bacteriocin in different levels of pH and temperatures were assessed. For the evaluation of the effect of temperature, bacterial cells of the target microorganisms were harvested as previously described. The working suspension composed of 1:1 bacterial cells of each test organism and individual bacteriocin were incubated for one hour in temperatures 4, 25, 30, 37, 45 and 60 °C. The effect of varying pH, on the other hand, was performed by re-suspending the collected biomass with 0.85% NaCl of pH 2.0, 4.0, 6.0, 8.0 and 10.0. The bacteriocin activity and adsorption for all the above-described set-up were assessed and quantified as previously described.

The members of the susceptible test panel were used as target organisms for the quantification of adsorption of bacteriocin in the presence of selected chemicals including NaCl, CaCl_2_, Tris, EDTA, K_2_HPO_4_, KH_2_PO_4_, Na_2_CO_3_ and Tween 80. Cells of each test organism were harvested as previously describe and re-suspended in 1% (*w*/*v*) solutions of each compound. Working solutions for each set-up were prepared to a final ratio of 1:1 (cell suspension: bacteriocin) and incubated at 37 °C for 1 h. The bacteriocin activity was assessed using a two-fold titration technique and the quantification of bacteriocin adsorption was calculated as previously described [39].

### 2.11. Bacteriocin Partial Purification

Two hundred milliliters of bacteriocin containing CFS were harvested from 18 h-old cultures of *E. faecium* strains ST651ea, ST7119ea and ST7319ea grown in MRS broth at 37 °C through centrifugation (4000× *g*, 30 min, 4 °C) which was subsequently subjected to heat inactivation (80 °C for 10 min) to eliminate heat-labile proteins. Protein precipitation was performed using ammonium sulfate, added in portions under continuous mixing overnight at 4 °C to obtain 60% saturation. Protein pellets were collected through centrifugation at 20,000× *g* for 1 h at 4 °C and were re-suspended in 20 mL of 100 mM potassium phosphate buffer (pH 6.5) and were separated via SepPakC_18_ hydrophobic column chromatography (Waters Millipore, Milford, MA, USA). Bacteriocin was eluted from SepPakC_18_ hydrophobic column with different concentrations (20%, 40%, 60% and 80%) of 2-propanol in 25 mM phosphate buffer (pH 6.5). Obtained protein fractions were filter sterilized using 0.20 μm syringe filters (Sartorius) and tested for bacteriocin activity against *L. monocytogenes* ATCC15313. Obtained sterile fractions were stored at −20 °C [40] for subsequent experiments.

### 2.12. Molecular Mass Estimation of Bacteriocins Using Tricine-SDS-PAGE

Concentrated bacteriocin obtained from ammonium sulfate precipitation (60% saturation) was subjected to tricine-SDS-PAGE following the protocol suggested by Schägger and Von Jagow [41]. A low-range rainbow molecular weight marker (Spectra low molecular range marker, Thermo Scientific) with sizes ranging from 3.4 kDa to 100 kDa was used for the size estimation. Two parts of the gel containing the bacteriocin loaded in mirror positions were simultaneously stained (with Coomassie blue R250) and overlayed with BHI agar (1% *w*/*v*, Difco) seeded with *L. monocytogenes* ATCC15313 (10^5^ CFU/mL) as the test strain to observe the location of inhibition zones for the size estimation of the active inhibitory proteins [42].

### 2.13. Evaluation of Bacterial Growth Inhibition of Test Organisms Using Semi-Purified Bacteriocin

Bacterial growth inhibition in growing conditions was assessed according to the method proposed by Todorov et al. [43]. Test organisms *L. monocytogenes* ATCC15313, *L. innocua* ATCC33090 and *E. faecium* VRE19 were grown individually in a sterile 96-well flat-bottom with the partially purified bacteriocin (60% 2-propanol fraction, obtained as described previously). Sterile BHI inoculated with 10% test organisms was dispensed in the first 10 columns of the plate, leaving the last two for sterility control and growth control. Each bacteriocin was added using concentrated (pure semi-purified sterile bacteriocin) and was diluted two-fold subsequently as the column number increases, wherein the highest bacteriocin activity used were as follows: ST651ea with 12,800 AU/mL, ST7119ea and ST7319ea with 25,600 AU/mL, wherein the working ratio of BHI inoculated with test organism and the designated bacteriocin is in 1:1 concentration. Experiments were performed in at least two independent set-ups for each bacteriocin, and test organism being treated. Plates were incubated at 37 °C for 24 h wherein bacterial turbidity was monitored at OD 600 nm for times 3, 6, 9 and 24 h.

### 2.14. Evaluation of Bacterial Cell Lysis of Non-Metabolically Active Test Organisms Using Semi-Purified Bacteriocins

Eighteen-hour old cultures of *L. monocytogenes* ATCC15313, *L. innocua* ATCC33090 and *E. faecium* VRE19 grown in BHI at 37 °C were harvested (4000× *g*, 30 min, 4 °C). The cells obtained were washed twice using 100 mM sterile potassium phosphate buffer (pH 6.5) and were re-suspended in the same buffer equaling to the original volume of the bacterial culture. Each bacterial suspension (80 µL) was individually dispensed on the sterile 96-well flat-bottom microtiter plates with 80 µL of designated concentrations (two-fold diluted) of an individual semi-purified bacteriocin according to recommendations of Todorov et al. [43]. Plates were incubated for 24 h at 37 °C wherein absorbance at OD 655nm was recorded at times 3, 6, 9 and 24 h. Bacterial cell lysis was quantified using the formula:

$$\%\;\mathrm{cell}\;\mathrm{lysis}=\left(100-\left(\frac{A_{t}}{A_{0}}\times 100\right)\right),$$

wherein A_t_ is the OD 655 nm reading at time 3, 6, 9 and 24 h after incubation, and A_0_ is the optical density reading before incubation (time 0).

### 2.15. Evaluation of Viable Cell Count Reduction Using Semi-Purified Bacteriocins

Test organisms in the early stationary phase (18 h-old cultures) *L. monocytogenes* ATCC15313, *L. innocua* ATCC33090 and *E. faecium* VRE19 grown in BHI at 37 °C were harvested at 4000× *g* at 4 °C for 15 min. The cells obtained were washed twice using sterile 1x PBS. Filter sterilized semi-purified bacteriocins (60% 2-propanol fraction) were added to a final proportion with the cell suspension at 1:1 with a total working volume of 3 mL. Viable bacterial cell count was obtained before and after incubation at 37 °C for 1 h by plating in BHI supplemented with 1.5% (*w*/*v*) agar. Wherein cell suspensions without any bacteriocin treatment were used as control.

## 3. Results

### 3.1. Isolation and Identification of Putative LAB Strains and Screening for Bacteriocin Production

A total of 180 putative LAB isolates were obtained from the locally produced fermented soybean paste (*doenjang*), all of which showed to be catalase-negative, were initially screened for antimicrobial activity against *L. monocytogenes* ATCC15313, and subsequently tested against *L. innocua* ATCC33090 and *E. faecium* VRE19. Eight out of 180 isolates showed inhibitory properties against the test organisms which were differentiated into three unique strains through DNA fingerprinting techniques (RAPD-PCR and repPCR) and all unique strains were identified to be Gram-positive, non-spore-forming, homofermentative coccus. The 16S rRNA sequencing analysis showed that the evaluated strains belong to the *Enterococcus faecium* species and were designated as strains *E. faecium* ST651ea, *E. faecium* ST7119ea and *E. faecium* ST7319ea.

### 3.2. Evaluation of Nature, Integrity and Stability of the Bioactive Molecule

The treatment of proteolytic enzymes abrogated the inhibitory activity of the CFS from all the strains. Furthermore, treatment of α-amylase was observed to have no significant impact on the activity of the CFS against the test organism *L. monocytogenes* ATCC15313. The stability of the bioactive molecules was further assessed by subjecting the crude CFS to commonly used substances in the food and feed production industries and by also subjecting these to varying temperatures (from 4 to 121 °C) and pH conditions (from pH 2 to pH 10). The bacteriocinogenic properties of *E. faecium* ST651ea, ST7119ea and ST7319ea were retained in the presence of EDTA, glycerol, milk, sodium chloride, Tween 20 and Tween 80 after incubation. Furthermore, the activity of crude CFS incubated in all temperatures and pH conditions retained their activity.

### 3.3. Bacteriocin Production, Growth Kinetics and Fermentation

**Figure 1:**
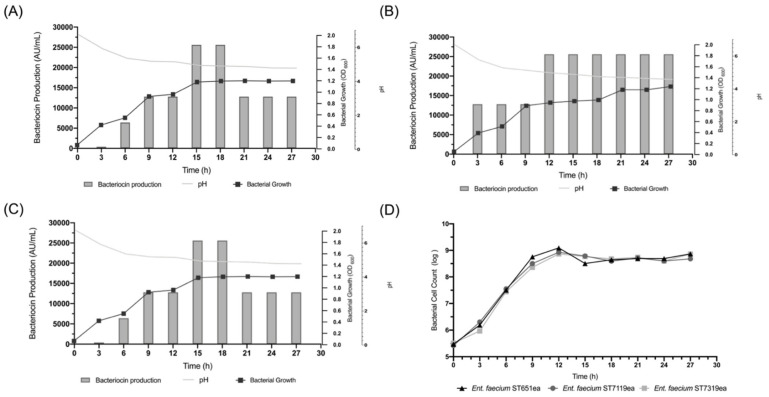
Bacterial growth, bacteriocin production and acidification of strains (**A**) *E. faecium* ST651ea, (**B**) *E. faecium* ST7119ea, (**C**) *E. faecium* ST7319ea and (**D**) corresponding bacterial cell count.

The bacterial cell density of the bacteriocinogenic cultures *E. faecium* ST651ea, ST7119ea and ST7319ea were observed (Figure 1) to have increased to approximately OD 600 nm 1.268, 1.240 and 1.198, respectively, showing robust growth in the culture medium and growth conditions used for this set-up. Quantification of bacterial cell growth, monitored by flow cytometry, showing that the number of bacterial cells increased up to log 8.86, 8.67 and 8.85, accordingly. Furthermore, the bacteriocin activity evaluated against *L. monocytogenes* ATCC15313, yielded up to 12,800 AU/mL, 25,600 AU/mL and 25,600 AU/mL from each strain, respectively, although a decrease in the bacteriocin activity profile was noted starting from time 21 h after inoculation for strain *E. faecium* ST651ea and time 24 h incubation for strain *E. faecium* ST7319ea. Subsequently, the pH after the 27 h incubation period was observed to drop to 4.3 for *E. faecium* ST651ea, 4.5 for *E. faecium* ST7119ea and 4.7 for *E. faecium* ST7319ea indicating a successful fermentative process for all the assessed bacteriocinogenic strains.

### 3.4. Effect of Varying Incubation Temperature on Bacteriocin Production of E. faecium ST651ea, ST7119ea and ST7319ea

Bacteriocin activity was observed to have decreased in half on both strains *E. faecium* ST651ea and ST7319ea when incubated at 45 °C for 18 h, wherein the maximum bacteriocin activity recorded on both strains were one level lower (6400 AU/mL and 12,800 AU/mL, respectively) than that of the optimal bacteriocin produced at 37 °C (Supplementary Figure S1). Bacteriocin activities in all other temperatures evaluated remained constant.

### 3.5. Screening for Bacteriocin Associated Genes

*E. faecium* ST651ea were found to harbor genes coding for both enterocin B and enterocin P, while strains *E. faecium* ST7119ea and *E. faecium* ST7319ea both have genes encoding for enterocin A and enterocin B. Subsequently, amplified PCR products were sequenced and analyzed to confirm identities and identify amino acid sequences of each gene present as shown in Table 2.

Comparison of the protein sequences between reconstructed amino acids and sequences from the database ADR70740.1 showed mutations in two amino acids on the bacteriocin produced by *E. faecium* ST651ea. Tryptophan and serine located on the N-terminal in the bacteriocin produced by *E. faecium* ST651ea were observed to be replaced by glycine and alanine. The same changes were determined in enterocin B produced by *E. faecium* ST7119ea. Moreover, enterocin B from *E. faecium* ST7119ea has a single amino acid mutation in the C-terminal, wherein lysine (basic amino acid) is replaced by asparagine (polar amino acid) (Table 2). On the other hand, enterocin P (Table 2) produced by *E. faecium* ST651ea has mutations on positions 33, 36 and 69 which were identified as glycine (neutral amino acid) was replaced with aspartic acid (acidic amino acid); valine (neutral amino acid) replaced by isoleucine (neutral amino acid), and isoleucine (neutral amino acid) replaced with methionine (polar amino acid), accordingly. In the case of *E. faecium* ST7319ea, the same mutations were also observed as in the previous strain but with additional mutations in positions 22 and 23, wherein tryptophan-alanine (neutral-neutral amino acids) were replaced with leucine-valine (neutral-neutral amino acids) (Table 2). Moreover, a comparison between the sequences of the enterocin A (as shown in Table 2) produced by *E. faecium* ST7119ea showed a high similarity with the reference sequence, but deletion of four amino acids in positions 57-58-59-60 (tyrosine-leucine-tyrosine-phenylalanine/polar-neutral-polar-neutral amino acids) was observed relative to the reference sequence (AP8904224.1). Additionally, only a single mutation cataloged by the deletion of alanine (neutral amino acid) was observed in *E. faecium* ST7319ea.

### 3.6. Bacterial Growth Inhibition (Cell Lysis) and Spectrum of Activity

**Figure 2:**
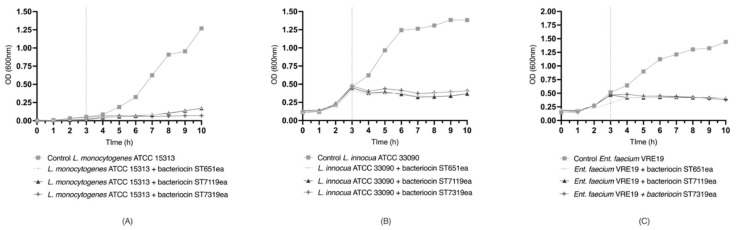
Bacterial growth inhibition of (**A**) *L. innocua* ATCC33090, (**B**) *L. monocytogenes* ATCC15313 and (**C**) *E. faecium* VRE19 by bacteriocins ST651ea, ST7119ea and ST7319ea.

Evaluation of bacterial growth inhibition by bacteriocins ST651ea, ST7119ea and ST7319ea against the test organisms *L. monocytogenes* ATCC15313, *L. innocua* ATCC33090 and *E. faecium* VRE19 conducted individually for each test organisms. After the addition of crude CFS with the activities 12,800 AU/mL for bacteriocin ST651ea and 25,600 AU/mL for both bacteriocin ST7119ea and bacteriocin ST7319ea during early-logarithmic phase (3 h after inoculation) showed a significant impedance in the growth of treated set-up at 4th h onwards comparative to the controls starting (Figure 2).

The range of inhibitory activity of bacteriocins studied was evaluated against the test panel as indicated in Table 3. Inhibitory activities of bacteriocins ST651ea, ST7119ea and ST7319ea were observed in most *Listeria* spp. and *Enterococcus* spp., but not representatives of *Staphylococcus* spp. and *Streptococcus* spp. from the test panel. Furthermore, beneficial strains except those that belong to the genus *Enterococcus* are all resistant to the evaluated bacteriocins.

### 3.7. Quantification of Adhered Bacteriocin on the Producer’s Cell Surface

A method suggested by Yang et al. [38] for extraction of attached bacteriocins on the surface of producer cells to potentially increase the concentration and the recovered antimicrobial peptides from the fermentation system, particularly for industrial application purposes. In this study, it was observed that considerable low amounts of bacteriocins (as shown in Supplementary Figure S3) have adhered to the surface of the producer organisms; wherein activities measured for strains *E. faecium* ST651ea was 100 AU/mL, while strains *E. faecium* ST7119ea and ST7319ea was 200 AU/mL.

### 3.8. Adsorption of Bacteriocin to the Target Cells

**Figure 3:**
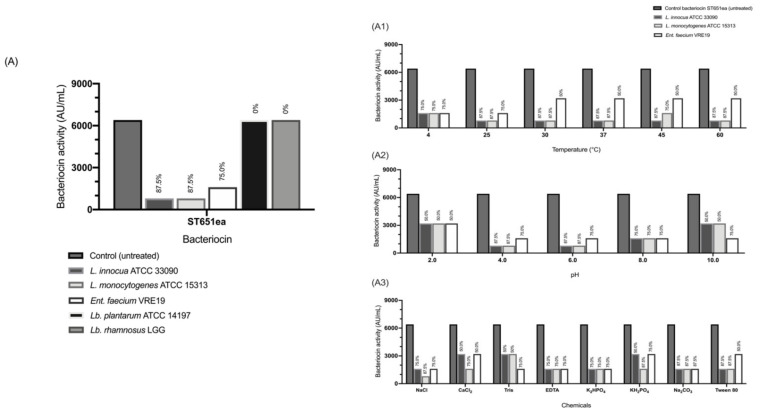

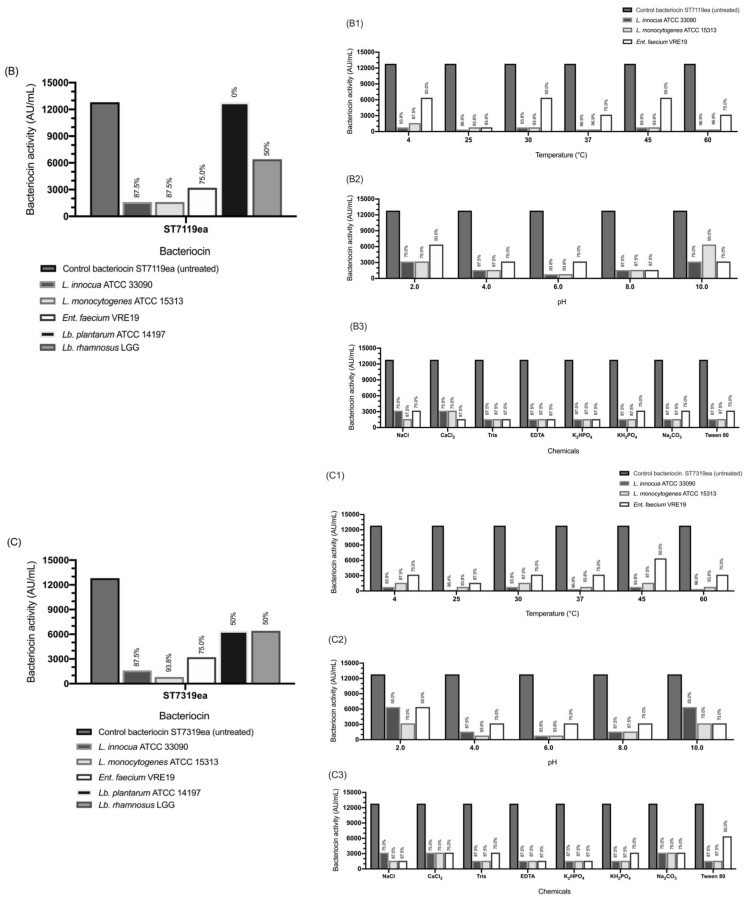
Adsorption rate (%) and bacteriocin activity of ST651ea after treatment to (**A**) target microorganisms and on sensitive test organisms under varying (**A1**) temperatures, (**A2**) pH and (**A3**) Addition of salts and organic compounds. Adsorption rate (%) of bacteriocin ST7119ea on (**B**) target microorganisms and absorption of bacteriocin in the surface of sensitive test organisms under varying (**B1**) temperatures, (**B2**) pH and (**B3**) addition of salts and organic compounds. Adsorption of bacteriocin ST7119ea on (**C**) target microorganisms and absorption of bacteriocin in the surface of sensitive test organisms under varying (**C1**) temperatures, (**C2**) pH and (**C3**) addition of salts and organic compounds.

The adsorption of bacteriocins to the target organisms evaluated through two groups of bacterial test panels showed that the adsorption rate of bacteriocin to sensitive test microorganisms (*L. monocytogenes* ATCC15313, *L. innocua* ATCC33090, *E. faecium* VRE19) was observed to be relatively higher (Figure 3A–C) compared to the non-susceptible ones considering that absorption rates ranging from 75.0% to 93.8%; whereas *Lb. plantarum* ATCC14197 and *Lb. rhamnosus* LGG have absorption rates of zero to 50.0% on all evaluated bacteriocins.

### 3.9. Factors Affecting the Adsorption of Bacteriocins to Sensitive Test Organisms

The effect of temperature was evaluated by bacteriocin treated metabolically inactive *L. monocytogenes* ATCC15313, *L. innocua* ATCC33090 and *E. faecium* VRE19 in temperatures 4, 25, 30, 37, 45 and 60 °C for 1 h, whereas the effect of pH using the same set of test organisms in non-growing condition was evaluated with the following pH conditions 2.0, 4.0, 6.0, 8.0 and 10.0 after an 1 h of incubation at 37 °C. Bacteriocin ST651ea was observed to have optimal adsorption to the target cells at temperatures 25 to 45 °C, wherein adsorption rates for *L. monocytogenes* ATCC15313 and *L. innocua* ATCC33090 were consistent at 87.5% except for *L. innocua* at 45 °C, in which adsorption decreased to a level lower (Figure 3(A1)). The maximum absorption of bacteriocin ST651ea to *E. faecium* VRE19 has been noted at 4 and 25 °C and consistent adsorption rate for temperatures 30 to 60 °C. For the evaluation of the effect of pH, it was observed that adherence of bacteriocin ST651ea to target cells optimally occurs between pH 4.0 to 8.0 for all test organisms (Figure 3(A2)). The same trends and tendencies were observed for bacteriocins ST7119ea and ST7319ea indicating that the optimal temperature for the bacteriocins produced by strains *E. faecium* ST651ea, ST7119ea and ST7319ea can effectively bind on the surface of the target organism, particularly the ones evaluated in this experiment, at the temperature range of 25 to 45 °C and pH conditions of 4.0, 6.0 and 8.0 (Figure 3(B1,B2,C1,C2)). The effect of inorganic compounds and salts, commonly used in the food production processes and other industrial applications were also assessed by subjecting the sensitive test organisms with the bacteriocins in the presence of NaCl, CaCl_2,_ Tris, EDTA, K_2_HPO_4_, KH_2_PO_4_, Na_2_CO_3_ and Tween 80 showing that a stable trend of bacteriocin adsorption occurs when present these compounds are present in the same system with the studied bacteriocins (Figure 3(A3,B3,C3)).

### 3.10. Bacteriocin Partial Purification

Bacteriocins ST651ea, ST7119ea and ST7319ea were precipitated from the CFS using 60% ammonium sulfate saturation, this allowed concentration of the active proteins produced during the fermentation prior to SepPakC_18_ hydrophobic chromatography separation with a step gradient of 20, 40, 60 and 80% 2-propanol in 25 mM phosphate buffer. Enterocins, known for their hydrophobic structure, were identified to be optimally obtained at 60% 2-propanol fraction in 25 mM phosphate buffer (pH 6.5) among the fractions used. Bacteriocin activity of semi-purified bacteriocins was observed to be at 51,200 AU/mL for bacteriocin ST651ea, 102,400 AU/mL for bacteriocin ST7119ea and 51,200 AU/mL ST7319ea against *L. monocytogenes* ATCC15313.

### 3.11. Molecular Size Estimation of Bacteriocins by Tricine-SDS-PAGE

As initially identified, bacteriocin genes present in *E. faecium* ST651ea were *ent*B and *ent*P while *E. faecium* ST7119ea and *E. faecium* ST7319ea both *ent*A and *ent*B. Results showed that the estimated sizes of antibacterial proteins expressed by strain *E. faecium* ST651ea, ST7119ea and ST7319ea have a median estimated size of 4.5 kDa which was confirmed by overlaying the unstained gel with *L. monocytogenes* ATCC15313 (as shown in Supplementary Figure S2).

### 3.12. Evaluation of Bacterial Growth Inhibition of Test Organisms Using Semi-Purified Bacteriocins

Test organisms *L. monocytogenes* ATCC15313, *L. innocua* ATCC33090 and *E. faecium* VRE19 growth inhibition were evaluated using the semi-purified bacteriocins (60% 2-propanol fraction). Each bacteriocin was evaluated in various concentrations for all the test organisms and bacterial cell density (OD 600 nm) was monitored for times 3, 6, 9 and 24 h after inoculation. For bacteriocin ST651ea, no significant change in the OD 600 nm reading was noted until the 9th h after incubation; the 24 h set-up showed that the minimum inhibitory concentration of all test organisms is at 800 AU/mL (Supplementary Figure S4A). Bacteriocins ST7119ea and ST7319ea, similarly, showed a slowed growth of bacterial cells until the 9th h. The minimum inhibitory concentration for semi-purified ST7119ea and ST7319ea are both 1600 AU/mL, one-fold lower compared to bacteriocin ST651ea (Supplementary Figure S4B,C).

### 3.13. Evaluation of Bacterial Cell Lysis in Non-Metabolically Active Test Organisms Using Semi-Purified Bacteriocins

Semi-purified bacteriocins (60% 2-propanol fraction) in different concentrations (two-fold dilution) were evaluated against *L. monocytogenes* ATCC15313, *L. innocua* ATCC33090 and *E. faecium* VRE19 in a non-dividing state for 24 h wherein the optical densities were monitored for time 3, 6, 9 and 24 h. The rate of cell lysis was calculated according to Todorov and Dicks [43]. The rate of bacteriocin-induced cell lysis for all bacteriocins tested was observed to share a similar trend for all test organisms. Most set-ups showed that the maximum cell lysis rate has been reached at time 3 h for both *L. monocytogenes* ATCC15313 and *E. faecium* VRE19, while *L. innocua* ATCC33090 was completely lyzed after 6 h of incubation in all bacteriocin titrations, except for bacteriocin ST7319ea (Supplementary Figure S5). Cell lysis of *L. monocytogenes* ATCC15313 treated with bacteriocin ST7319ea showed slower and lower cell lysis starting at 200 AU/mL as compared to the other two bacteriocins, these differences show the distinction between the two enterocin A and B producing strains albeit sharing the same type and activity of bacteriocins produced (Supplementary Figure S5C) [44,45,46,47].

### 3.14. Quantification of Viable Cells of Test Organisms after Treatment of Semi-Purified Bacteriocins

Test organisms *L. monocytogenes* ATCC15313, *L. innocua* ATCC33090 and *E. faecium* VRE19 were all tested against semi-purified bacteriocins ST651ea, ST7119ea and ST7319ea (60% 2-propanol fraction) at a ratio 1:1 with the bacterial cell suspension of the test organisms. Initial bacterial cell populations were determined to be 2.0 × 10^9^, 3.0 × 10^9^ and 3.0 × 10^9^ CFU/mL, respectively, and the viable cell counts were quantified by spot plating (10 µL) after an hour of incubation. No viable cells were detected in both *L. monocytogenes* ATCC15313 and *E. faecium* VRE19 treated with all the bacteriocins (data not shown). While a single-cell growth of *L. innocua* ATCC33090 (100 CFU/mL) was detected when treated with bacteriocin ST651ea, but no cell growth was observed in cells treated with bacteriocins ST7119ea and ST7319ea (data not shown). These data support that the bacteriocins being studied are bactericidal, whose mechanism of action illustrates bacterial cell lysis.

## 4. Discussion

*Enterococcus* spp. are known for their ability to survive a broader range of pH compared to the other LAB. This aided in their wide ecological distribution, particularly their association with human and other animal gut microbiota [48,49]. Furthermore, members of this genus are known to be autochthonous and are associated with the phyllosphere of plants allowing them to play a significant role in the ripening of various plant-based fermented food products including *doenjang*, a Korean traditional fermented soybean paste [50]. Additionally, the widespread association of bacteriocinogenic enterococci on *miso*-paste (a soybean-based fermented food product from Japan) was identified by Onda et al. [51], denoting that *E. faecium* were the dominant bacteriocinogenic LAB isolated from a soybean-based fermented food source. Consequently, it was postulated that the presence of various bacteriocinogenic enterococci in these types of fermented food products acts as an additional hurdle against spoilage microorganisms. The bacteriocins produced by some strains of *E. faecium* and *E. faecalis* have also been applied as biocontrol agents of contaminants and spoilage organisms in food and feed products [52,53,54] and human and other animal relevant pathogens [4,16,22,26,55,56].

Bacteriocins, primarily identified through their proteinaceous nature, are active proteins against bacteria that are taxonomically close to the producer strains [11,28]. However, aside from this, various antimicrobial by-products are also associated with LAB which has been majorly linked to the fermentation pathway. The concomitant of low pH by the accumulation of organic acids has been associated with the bacterial contaminant inhibitory properties along with other antimicrobial by-products such as hydrogen peroxide, carbon dioxide, diacetyl and low molecular weight proteins [8,48]. In this study, ruling out the other probable inhibitory bioactive substances by pH neutralization and thermal treatment, and the abrogation of antibacterial activity against *L. monocytogenes* ATCC15313 when crude CFS were treated with proteolytic enzymes and hydrolytic enzyme indicate that the bioactive inhibitory molecule is proteinaceous without associated carbohydrate moiety on its structure.

The stability of bacteriocins produced by *Enterococcus* spp., particularly enterocins, are long been studied and assessed for their application as food additives for biocontrol and specific infection treatment due to their stability and narrow-spectrum [9,11]. According to Dillon [57], the structural conformation of Class II bacteriocins allows them to be stable in wide fluctuations of environmental temperature and pH. As observed, the bacteriocins studied were stable in all pH conditions and temperatures. Additionally, no significant effect of the presence of the selected chemicals was observed on the activity of all the bacteriocins evaluated. These provide valuable information on the probable structure and further possible applications of the studied bacteriocins especially as agents for biocontrol of specific contaminants in food and feed production and the clinical settings.

Enterocins are known to have potent anti-listerial activity. Some of these well-characterized anti-listerial enterocins were from bacteriocinogenic strains isolated from Spanish dry fermented sausage [32], artisanal cheese [58,59], smoked salmon [60], infant feces [61,62], vaginal secretions [63], monkeys [64] and wild mallard [65]. In this study, bacteriocinogenic strains *E. faecium* ST651ea, ST7119ea and ST7319ea, all isolated from fermented soybean paste, were measured and quantified for their bacteriocin activity against *L. monocytogenes* ATCC15313 along with the changes in cell density, pH and corresponding cell counts (Figure 1A–D). As observed, a decrease in bacteriocin activities of both ST651ea and ST7319ea occurred. This phenomenon can be due to the degradation of the antibacterial proteins produced by these strains by proteolytic enzymes released in the system when the cells entered the death phase as suggested by Todorov and Dicks [66].

From a different perspective, the use of live microorganisms in food systems as biocontrol has long been used in industries. The observed decrease in bacteriocin activity at 45 °C incubation is due to the decreased bacterial growth compared to the other set-ups. As described by Gardini et al. [67], causes of decrease in the production of metabolites are directly correlated to the bacterial growth in the system, and temperature changes are considered as the primary factor. Furthermore, it was mentioned by Yang et al. [68] that temperature beyond the optimal growth condition contributes to a longer lag phase, thus affecting the bacterial growth rate and final cell density.

Initial screening for the bacteriocin associated genes was performed through PCR using primers amplifying regions of enterocin encoding genes (*ent*A, *ent*B, *ent*L50B and *ent*P) and additional bacteriocin genes associated with nisin and pediocin PA-1, both were atypically identified to be present in some bacteriocinogenic *Enterococcus* as demonstrated by Todorov et al. [69]. The presence of *ent*A, *ent*B and *ent*P were collectively identified for all the strains evaluated. Comparative analysis of reconstructed sequences from the identified bacteriocin-associated genes shown notable mutations as indicated in Table 2. Mutations observed in enterocin B produced by all the evaluated strains were observed to be substitutions within and at the C-terminal of the peptide sequences. Since it has been established that the N-terminal of antimicrobial peptides plays a crucial role in the recognition of the receptors in the surface of the target microorganism, this then indicates that the observed mutations in the enterocin B produced by *E. faecium* ST651ea and ST7319ea do not affect the spectrum of activity but may play a significant role on their mode of actions [28]. Alterations in the amino acid sequences of enterocin P produced by *E. faecium* ST651ea were similar to the observed changes in enterocin B, thus this does not concern the spectrum of activity but may affect its mode of action.

Bacterial growth of *L. monocytogenes* ATCC15313 and *L. innocua* ATCC33090 treated with each bacteriocin have similar patterns as previously observed from bacteriocins produced by other LAB [12,21,34,69,70]. Evidence showing that bacteriocins can be a potential treatment against VRE in vitro and in vivo was demonstrated by Kim et al. [71] using a four-bacterium consortium including *Blautia producta*, confirmed to be carrying a nisin-like encoding gene. Enterocins identified from the studied strains are classified under class II, whose general mechanism of action causes cell lysis. The attachment of active peptides to the specific docking region causes the channel of sugar permease to remain on its active (open) conformation leading to leakage of extracellular matrix components, which eventually leads to cell death [12]. This information also determines the spectrum of target organisms where the bacteriocins can be active.

Identifying the range of activity of the studied bacteriocin is imperative to assess their possible applications. The test panel generally consisted of LAB—some of which are previously characterized for their beneficial properties as probiotics, food-borne emerging and non-emerging pathogens and clinical isolates of VRE were used. The narrow spectrum of activity of bacteriocins, in general, compared to antibiotics made them advantageous for targeted treatments or biocontrol [72] particularly with the continuous emergence of multi-drug resistant pathogens. A sec-dependent bacteriocin, Bac32, characterized by Inoue et al. [26] have specific activity against *Enterococcus* species including *E. faecium*, *E. hirae* and *E. durans*, but not against *L. monocytogenes* demonstrates the possible applications of bacteriocins that can be revolutionary particularly on using the approach of precision medicine in treating infections caused by emerging resistant pathogens.

As proposed by Yang et al. [38], a potential increase in bacteriocin yield can be achieved by decreasing environmental pH causing liberation of attached bacteriocins to the producer cells’ surface. In this study, the observations noted for *E. faecium* ST651ea, ST7119ea and ST7319ea coincide with previously characterized bacteriocinogenic LAB [56,66] showing that only relatively small amounts of bacteriocins have adhered to the cell surface of the producer organism.

To use these bacteriocins effectively, especially as a suggested alternative for the treatment of emerging and re-emerging pathogens particularly those that harbor antibiotic resistance, understanding the factors that affect the mode of attachment and their adsorption is vital [73,74,75,76]. In this study, higher rates of absorption were demonstrated on the susceptible panel in contrast with the non-susceptible test microorganisms. These observations coincide with the trends indicated by Todorov [75] on bacteriocin AMA-K, indicating that the adsorption of bacteriocins to the susceptible cells ranges between 70% to 100%, while resistant cells have a lower range of adsorption (20%). The high adsorption of studied bacteriocins to the evaluated pathogenic strains, emphasizes the advantage of the use of bacteriocins in an environment comprised of diverse microorganisms due to its specificity. Additionally, the environmental conditions in which bacteriocins are employed play a significant role in their mode of action(s) thereby affecting their efficiency in inhibiting target organisms present in a system. Due to their nature, bacteriocins can be affected by fluctuations of temperature and pH by interfering with the integrity of their structures, subsequently hindering their activity [77,78]. Experimental observations showed that the adsorption of bacteriocins being studied is stable in fluctuations of environmental conditions, wherein the observed trends and tendencies coincide with various studies previously conducted [56,68,74,75,79]. It was mentioned by De Vuyst et al. [80] that the rate of decrease of bacteriocin activity is not temperature-dependent and is more evident when alterations in environmental pH occur as they have modeled through a batch fermentation set-up. Furthermore, a method proposed by Yang [38] that bacteriocin adsorption on the cell surface decreases as pH leans to an acidic range, this scenario does not exclude the target cells, supporting the decrease of bacteriocin adsorption as the pH decreases. These observations were supported by the study conducted by Yildirim et al. [74] wherein demonstration of the adsorption of buchnericin LB were optimally observed between pH 5.0 to 8.0, whereas below or above the mentioned range showed a decreased adsorption rate. Various mechanisms on the decrease of activity beyond this pH range can be responsible for the increase of residual bacteriocin to the environment such as protein aggregation, degradation or structure denaturation [80]. Furthermore, Yildirim et al. [74] shown that salts cause a decrease in adsorption of buchnericin LB to the sensitive test organisms while Na_2_CO_3_ and EDTA do not have significant effects on the adsorption of bacteriocins. These findings coincide with the data presented in this study, indicating the stability of studied bacteriocins in all the assessed compounds.

Partial purification of the bacteriocins expressed was carried out to further confirm the identity of the bacteriocins being expressed by the studied strains. Observations from the tricine-SDS-PAGE conducted showed expressed proteins have similar molecular sizes in comparison to the previously characterized bacteriocins produced by *Enterococcus* spp. including *E. faecium* ST62BZ [81], *E. faecium* CTC492 [32] and *E. faecalis* FA2-2 (pYI17) [82]. Generally, enterocins have an estimated size of ≤6 kDa the same as most members of the Class II bacteriocins [34,43,83,84].

Even though there are multiple mechanisms of action associated with this group of bacteriocins from LAB, it has been found out that the general mechanism involves the outer cell membrane particularly the sugar uptake/phosphorylatory system which serves as a docking molecule in *Listeria* spp., which has been further supported by the emergence of bacteriocin-resistant mutants [85]. To confirm the mechanism of actions of the expressed bacteriocins, activities were assessed in both metabolic and metabolically inactive cells, wherein observations on the inhibition of bacterial growth of *L. monocytogenes* ATCC15313, *L. innocua* ATCC33090 and *E. faecium* VRE19 supports this hypothesis. Additionally, rates of cell lysis in the non-metabolic set-up further confirms the stated mechanisms of the enterocins. However, the observed variations in the rates of cell lyses between the *Listeria* sp. supports strain-level differences among isolated bacteriocins in *E. faecium* [78]. Moreover, the quantification of viable cells treated with semi-purified bacteriocins further supports these observations.

## 5. Conclusions

*Doenjang*, a Korean traditional fermented soybean paste, was found to harbor multiple strains of *E. faecium*, consequently are expressing functional properties including bacteriocin production. In this study, the partial characterization of the bacteriocins produced by the strains *E. faecium* ST651ea, *E. faecium* ST7119ea and *E. faecium* ST7319ea presented possible applications in the control of pathogens including *L. monocytogenes*, *L. innocua* and vancomycin-resistant *Enterococcus.* Based on the applied experimental approaches (sensitivity to proteolytic enzymes, partial purification by precipitation with ammonium sulfate, separation by SepPakC18 hydrophobic chromatography and Tricine SDS-PAGE) allows the confirmation of the proteinaceous nature of the antimicrobial agent/s produced by studied strains. Furthermore, bacteriocin structural genes PCR targeting assay shows evidence for possible production of more than one bacteriocin for each strain, indicated by the presence *ent*B and *ent*P genes for *E. faecium* ST651ea, while both *E. faecium* ST7119ea and *E. faecium* ST7319ea harbor *ent*A and *ent*B genes. Thus, there is a delimitation on the identification of the expressed bacteriocins based on the applied partial purification of the presumed expressed antimicrobial proteins (enterocin A, enterocin B and enterocin P) due to their close molecular sizes. Additionally, variations in the obtained spectrum of activity allows postulation of variabilities in the bacteriocins expressed by the strains. Therefore, a future study involving the expression of the studied bacteriocin on the RNA level and/or appropriative purification, amino-acid sequencing and mass spectrometry analysis should be carried out to further address this question to properly discriminate which bacteriocin(s) is(are) expressed by the strains. However, the narrow spectrum and specificity of the bacteriocins evaluated, along with their stability in the alterations of environmental conditions can be an advantage for their possible future applications in the food and feed industries and can also be employed in precision medicine (infection associated treatments) in both veterinary and clinical setting. However, it is recommended that evaluation of safety and screening for additional beneficial metabolites should be performed before industrial applications.

## Figures and Tables

**Table 1:** Primers used in this study for the detection of bacteriocin-associated genes.

Bacteriocin	Primer	Oligonucleotide Sequence (5′–3′)	Product Size (bp)	References
Enterocin A	entA-F	GAG ATT TAT CTC CAT AAT CT	452	[32]
	entA-R	GTA CCA CTC ATA GTG GAA		
Enterocin B	entB-F	GAA AAT GAT CAC AGA ATG CCT A	159	[33]
	entB-R	GTT GCA TTT AGA GTA TAC ATT TG		
Enterocin L50B	entL50B-F	ATG GGA GCA ATC GCA AAA TTA	135	[34]
	entL50B-R	TAG CCA TTT TTC AAT TTG ATC		
Enterocin P	entP-F	ATG AGA AAA AAA TTA TTT AGT TT	216	[35]
	entP-R	TTA ATG TCC CAT ACC TGC CAA ACC		
Nisin	Nis-F	ATG AGT ACA AAA GAT TTCAAC TT	203	[36]
	Nis-R	TTA TTT GCT TAC GTG AAC GC		
Pediocin	PedPro-F	CAA GAT CGT TAA CCA GTT T	1238	[37]
	Ped1041-R	CCG TTG TTC CCA TAG TCT AA		

**Table 2:** Detected bacteriocin genes and predicted protein sequences.

Strain ID	Identified Bacteriocin Genes		Protein Sequences
*E. faecium* ST651ea	*ent*B	Reconstructed sequences	ENDHRMPNELNRPNNLSKG**GA**KCGAAIAGGLFGIP KGPLA WAAGLANVYS KCN
		GenBank: ADR70740.1	**WS**KCGAAIAGGLFGIPKPLAWAAGLANVYSKCK
	*ent*P	Reconstructed sequences	MRKKLFSLTL IGKFGLVVTN FGTKVDAATR SY**D**NG**I**YCNNS KCWVNWGEAKE NIAGIVISGW ASGLAG**M**GH
		GenBank: ERK34332.1	MRKKLFSLTL IGKFGLVVTN FGTKVDAATR SY**G**NG**V**YCNNS KCWVNWGEAKENIAGIVIS GWASGLAG**I**GH
*E. faecium* ST7119ea	*ent*A	Reconstructed sequences	SKDPKYSDI LEVLQKVYLK LEKQKYELDP GPLINRLVN**- - - -**TAYTN KIRFTEYQEELIRNLSEIGRT AGINGLYRA DYG
		GenBank: AP8904224.1	MKKNAKQIVH ELYNDISISK DPKYSDILEV LQKVYLKLEKQ KYELDPGPLI NRLVN**YLYF**T AYTNKIRFTE YQEELIRNLSE
	*ent*B	Reconstructed sequences	ENDHRMPNELN RPNNLSKG**GA**KCGAAIAGGLFGI PKGPLAWAAGLANVYSKC**N**
		GenBank: ADR70740.1	**WS**KCGAAIAGGLFGI PKGPLAWAAGLANVYSKC**K**
*E. faecium* ST7319ea	*ent*A	Reconstructed sequences	GSAK MKKNAKQIVHELYNDISISKDPKYSDILEVL QKVYLKLEKQKYELDPGPLINRLVNYLYFT**-**YTN KIRFTEYQEELIRNLSEIGRTAGINGLYRADYGE
		GenBank: AP8904224.1	MKKNAKQIVHELYNDISISKDPKYSDILEVL QKVYLKLEKQKYELDPGPLINRLVNYLYFT**A**Y TNKIRFTEYQEELIRNLSE
	*ent*B	Reconstructed sequences	ENDHRMPNELNRPNNLSKG**GA**KCGAAIAG GLFGIPKGPLA**LV**AGLANVYSKC**N**
		GenBank: ADR70740.1	**WS**KCGAAIAGGLFGIPKGPLA**WA**AGLANVYSKC**K**

Observed differences were indicated in blue, bold, underlined letters.

**Table 3:** The spectrum of antibacterial activity of bacteriocins produced by strains *E. faecium* ST651ea, ST7119ea and ST7319ea.

Test Organism	Number of Strains with Positive Inhibition/Total Number of Strains		
	*E. faecium* ST651ea	*E. faecium* ST7119ea	*E. faecium* ST7319ea
***Enterococcus* spp.**			
*E. avium* ^c^	0/3	0/3	0/3
*E. durans* ^c^	0/1	0/1	0/1
*E. gallinarum* ^c^	0/1	0/1	0/1
*E. faecium* ^c^	**10**/12	**12**/12	**11**/12
*E. faecalis* ^c^	**2**/2	**2**/2	**2**/2
*E. thailandicus* ^c^	**1**/1	**1**/1	**1**/1
**Vancomycin-resistant *Enterococcus* spp.**			
*E. lactis* ^d^	**1**/1	**1**/1	0/1
*E. faecium* ^d^	**9**/11	**7**/11	5/11
VRE clinical isolates ^d^	**15**/19	**18**/19	**19**/19
***Lactobacillus* spp.**			
*Lb. brevis* ^c^	0/3	0/3	0/3
*Lb. coryniformis* ^c^	0/1	0/1	0/1
*Lb. curvatus* ^c^	0/2	0/2	0/2
*Lb. fermentum* ^c^	0/2	0/2	0/2
*Lb. paracasei* ^c^	**1**/5	**1**/5	**1**/5
*Lb. plantarum* ^ac^	0/9	0/9	0/9
*Lb. reuteri* ^c^	0/2	0/2	0/2
*Lb. rhamnosus* ^c^	0/5	0/5	0/5
*Lb. rhamnosus* LGG ^a^	0/1	0/1	0/1
*Lb. sakei* ^c^	**1**/5	**1**/5	**1**/5
*Lb. salivarius* ^c^	0/3	0/3	0/3
***Lactococcus* spp.**			
*Lc. lactis* ^c^	0/2	0/2	0/2
***Leuconostoc* spp.**			
*Leuc. citreum* ^c^	0/1	0/1	0/1
*Leuc. mesenteroides* ^ce^	0/7	0/7	0/7
***Listeria* spp.**			
*L. monocytogenes* ^a^	**1**/1	**1**/1	**1**/1
*L. innocua* ^a^	**1**/1	**1**/1	**1**/1
***Pediococcus* spp.**			
*P. acidilactici* ^ce^	0/4	0/4	0/4
*P. pentosaceus* ^c^	**1**/3	0/3	0/3
***Staphylococcus* spp.**			
*S. arlettae* ^b^	0/1	0/1	0/1
*S. aureus* ^b^	0/1	0/1	0/1
*S. auricularis* ^b^	0/1	0/1	0/1
*S. capitis* subsp. *capitis* ^b^	0/1	0/1	0/1
*S. carnosus* subsp. *carnosus* ^b^	0/1	0/1	0/1
*S. cohnii* subsp. *cohnii* ^b^	0/1	0/1	0/1
*S. delphini* ^b^	0/1	0/1	0/1
*S. epidermidis* ^b^	0/2	0/2	0/2
*S. haemolyticus* ^b^	0/1	0/1	0/1
*S. hominis* ^b^	0/1	0/1	0/1
*S. aureus* MRSA ^ad^	0/6	0/6	0/6
*S. lentus* ^b^	0/1	0/1	0/1
*S. succinus* ^b^	0/1	0/1	0/1
*S. warneri* ^b^	0/1	0/1	0/1
***Streptococcus* spp.**			
*Str. gordonii* ^b^	0/1	0/1	0/1
*Str. mitis* ^b^	0/1	0/1	0/1
*Str. mutans* ^b^	0/1	0/1	0/1
*Str. sanguinis* ^b^	0/1	0/1	0/1
*Str. salivarius* subsp. *Salivarius* ^b^	0/1	0/1	0/1
*Str. sanguinis* ^b^	0/1	0/1	0/1
*Str. thermophilus* ^b^	0/1	0/1	0/1
***Weisella***			
*W. cibaria* ^c^	0/1	0/1	0/1

Positive inhibitions were presented in bold numbers and sources of the bacterial strains evaluated are as follows (^a^) American Type Culture Collection (ATCC); (^b^) Korean Agricultural Culture Collection (KACC); (^c^) HEM Culture Collection; (^d^) Laboratory of Antimicrobials; (^e^) ProBacLab Culture Collection.

## Supplementary Materials

The following are available online at https://www.mdpi.com/article/10.3390/microorganisms9051085/s1, Figure S1: Identification of bacteriocin production of strains *Ent. faecium* ST651ea, *Ent. faecium* ST7119ea and *Ent. faecium* ST7319ea in varying temperatures; Figure S2: Tricin-SDS-PAGE gel image of the bacteriocins expressed by *E. faecium* strains ST651ea, ST7119ea and ST7319ea (A) stained by Coomasie-blue R250 and (B) confirmation of the location and size estimation of the expressed proteins on the gel overlayed with BHI inoculated with *L. monocytogenes* ATCC15313. Figure S3: Measured (A) adhered bacteriocins to the producer cells’ surface as shown in comparison with the (P) produced bacteriocin quantified against *L. monocytogenes* ATCC15313.; Figure S4: Effect of semi-purified bacteriocins (A) ST651ea, (B) ST7119ea and (C) ST7319ea on the growth of *Listeria innocua* ATCC33090, *Listeria monocytogenes* ATCC15313 and *Ent. faecium* VRE19.; Figure S5: Rate of cell lysis (%) of non-metabolically active *Listeria innocua* ATCC33090, *Listeria monocytogenes* ATCC15313 and *Enterococcus faecium* VRE19 treated with various concentrations of partially purified (A) bacteriocin ST651ea, (B) bacteriocin ST7119ea and (C) bacteriocin ST7319ea (on periods 3 h, 6 h, 9 h and 24 h).
