# Supplementary material for: Characterization of Partially Purified Bacteriocins Produced by Enterococcus faecium Strains Isolated from Soybean Paste Active Against Listeria spp. and Vancomycin-Resistant Enterococci
Source: Microorganisms. 2021 May 18;9(5):1085. doi: 10.3390/microorganisms9051085 (PMC8158364; doi:10.3390/microorganisms9051085)
Supplement: Supplementary file 1 [file microorganisms-09-01085-s001.zip › Supplementary Material.pdf]

Supplementary Material

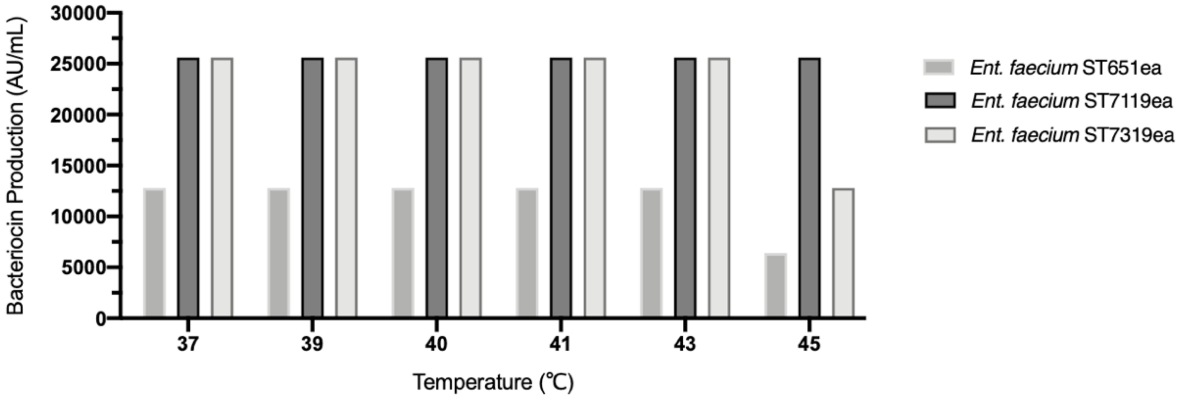

Figure S1. Identification of bacteriocin production of strains *Ent. faecium* ST651ea, *Ent. faecium* ST7119ea, and *Ent. faecium* ST7319ea in varying temperatures.

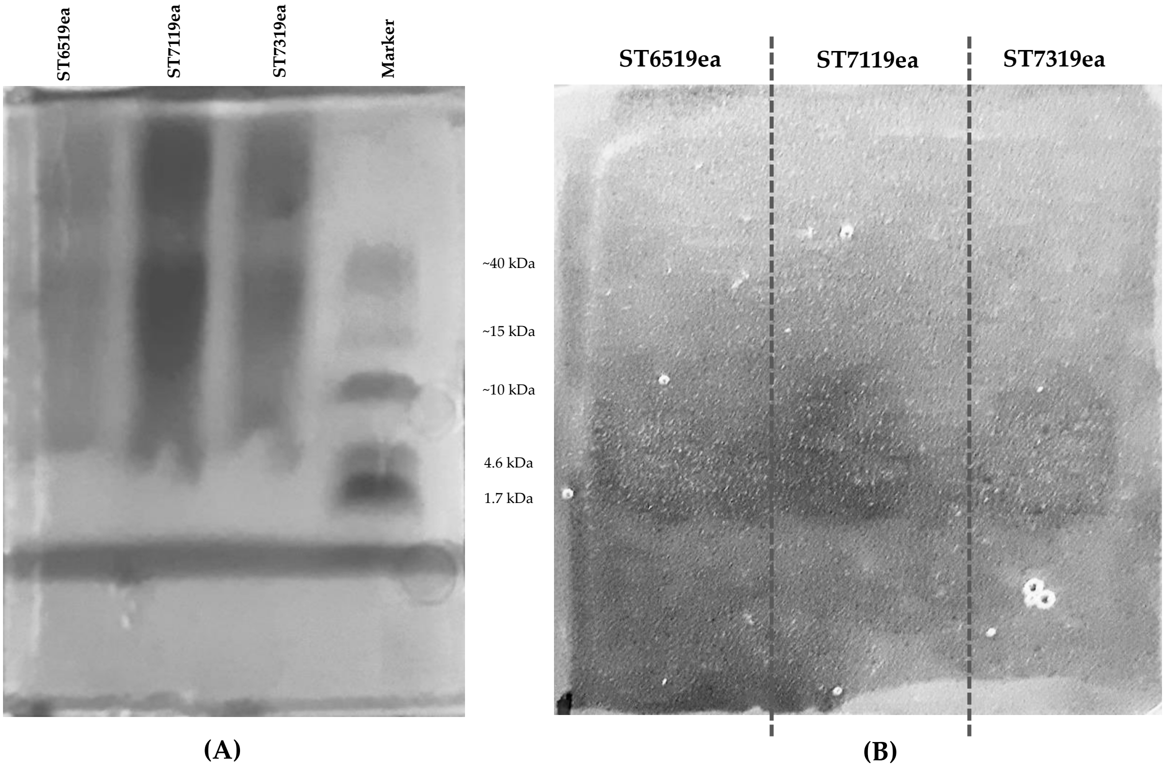

Figure S2. Tricine-SDS-PAGE images of bacteriocins expressed by *E. faecium* strains ST651ea, ST7119ea, and ST7319ea **(A)** strained by Coomasie-blue R250 and **(B)** confirmation of the location and size estimation of the expressed proteins on the gel overlayed with BHI inoculated with *L. monocytogenes* ATCC15313.

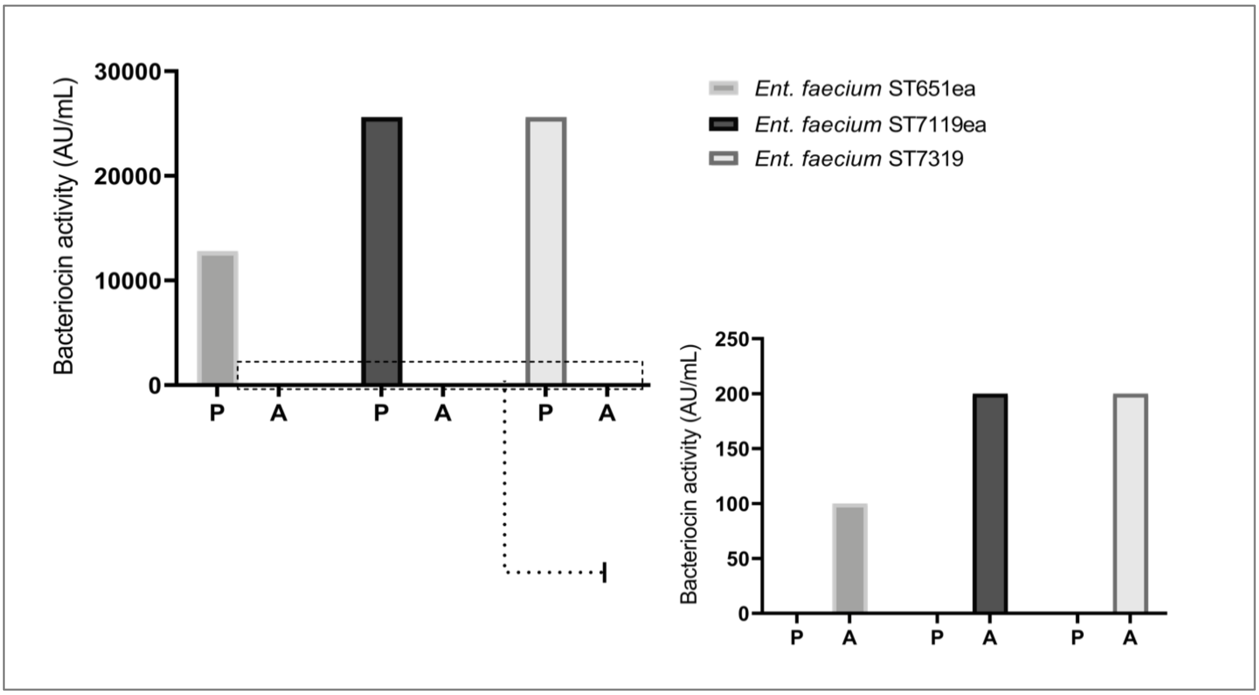

Figure S3. Measured **(A)** adhered bacteriocins to the producer cells' surface as shown in in comparison with the **(P)** produced bacteriocin quantified against *L. monocytogenes* ATCC15313.

(A) Bacteriocin ST651ea

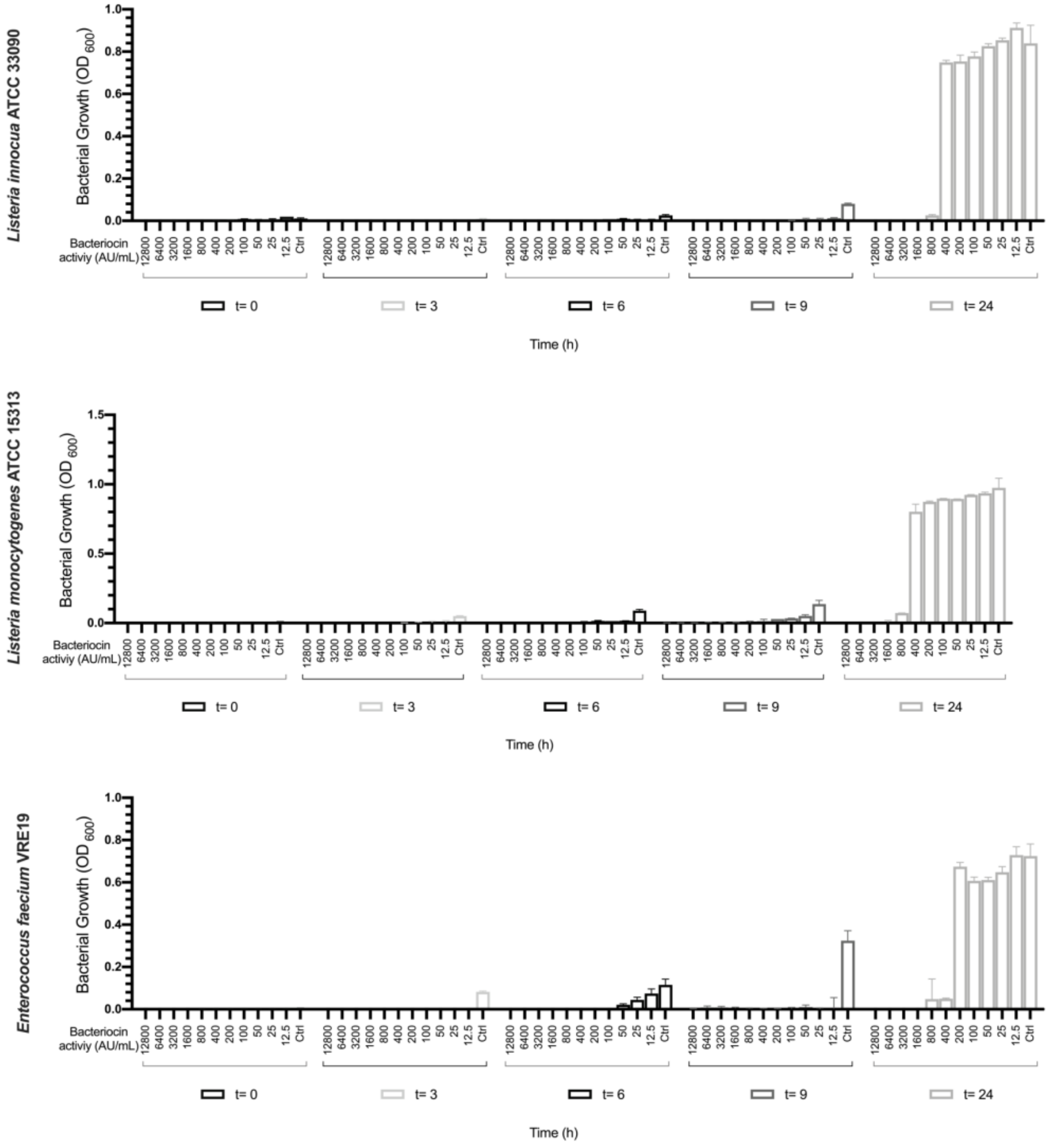

(B) Bacteriocin ST7119ea

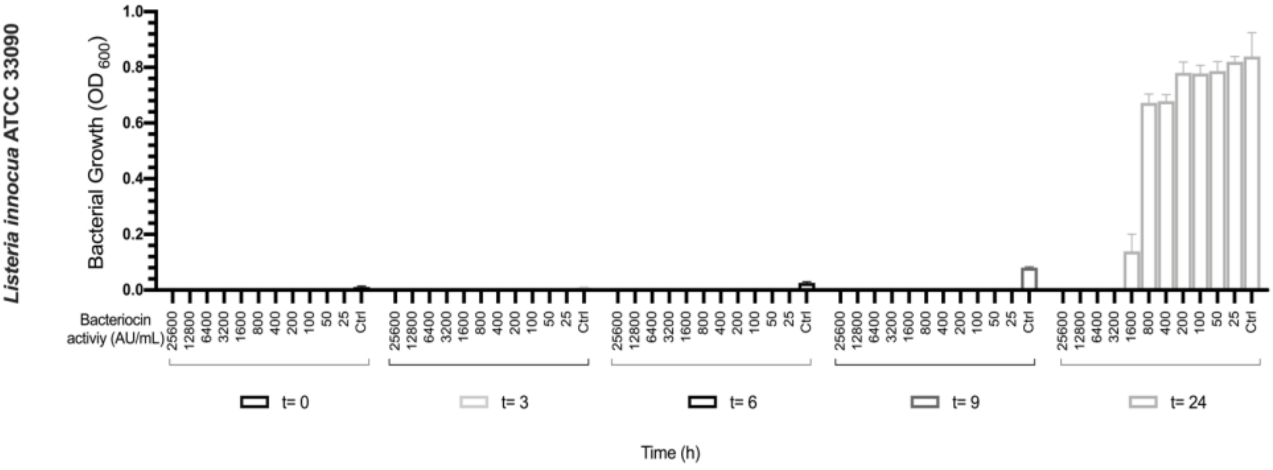

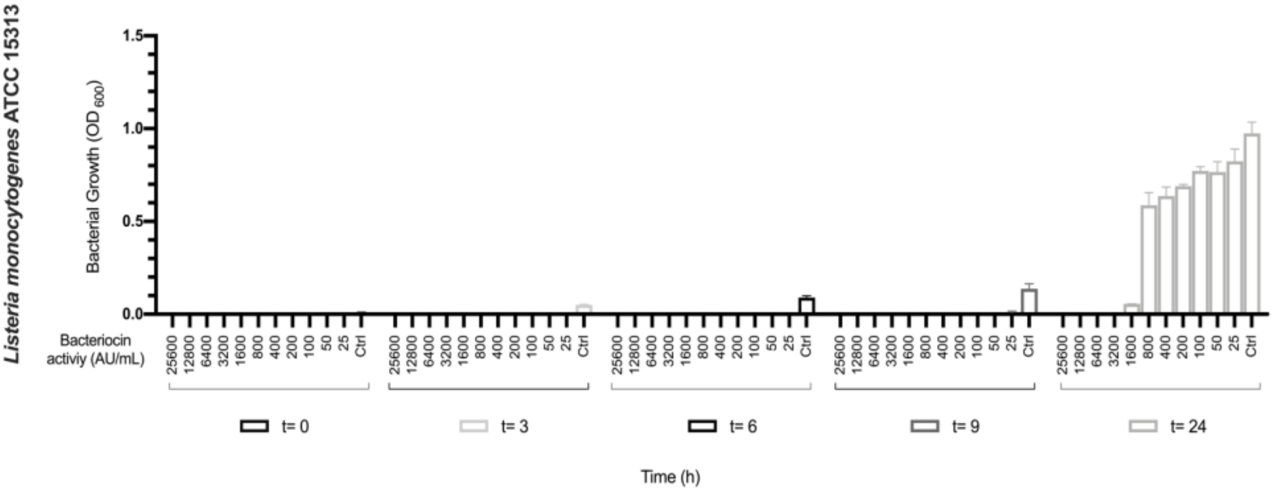

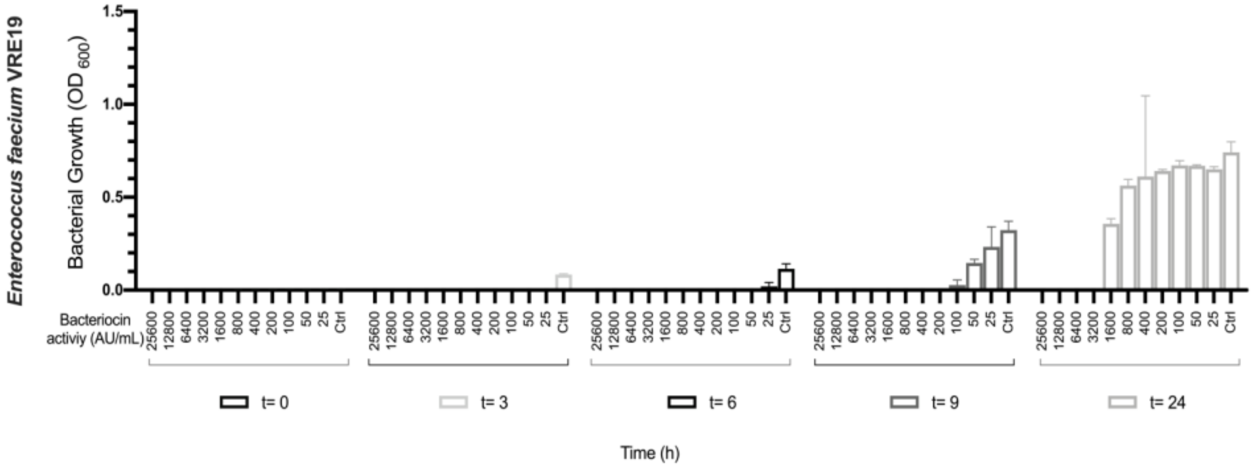

(C) Bacteriocin ST7319ea

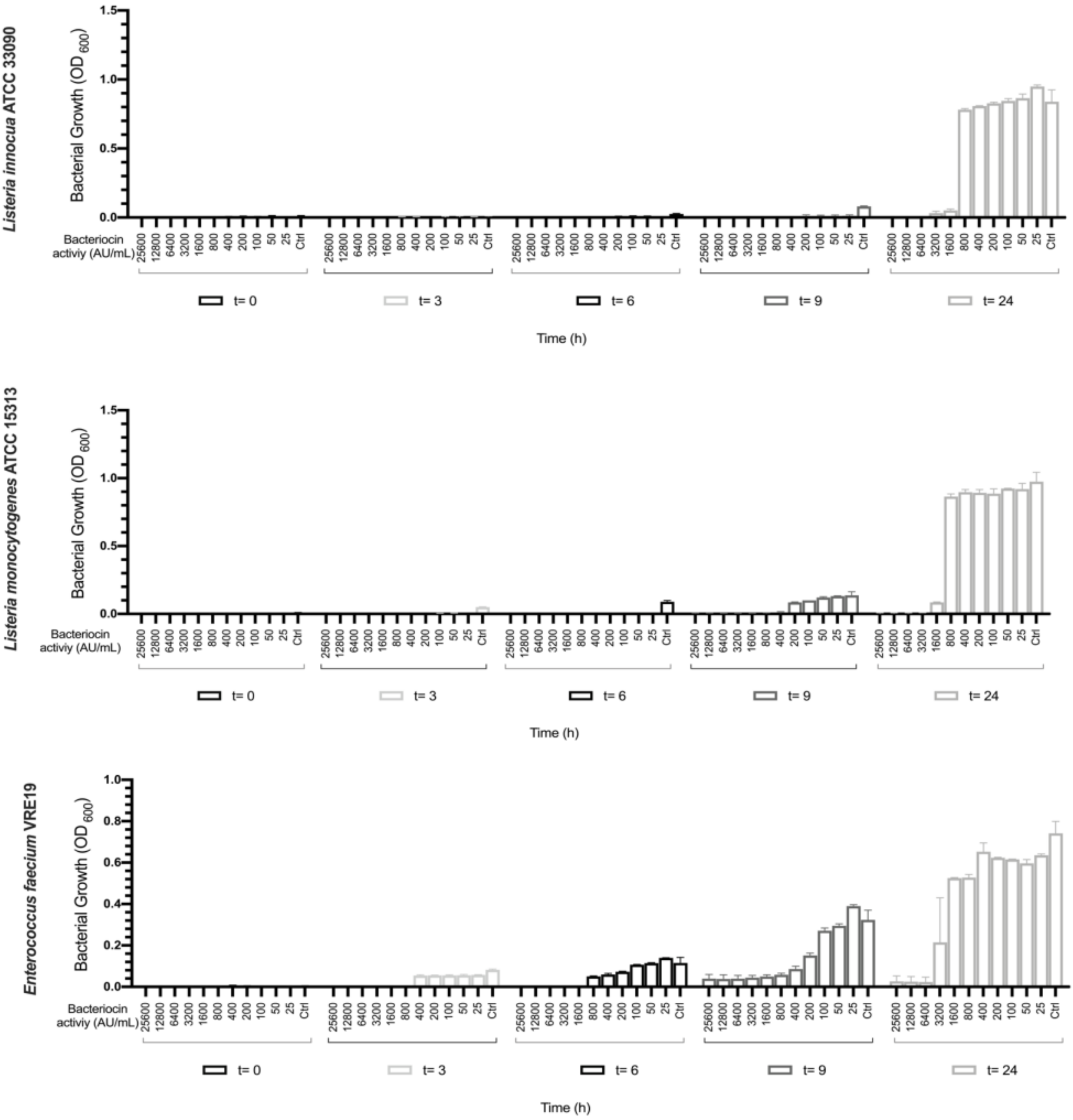

Figure S4. Effect of partially purified bacteriocins **(A)** ST651ea, **(B)** ST7119ea, and **(C)** ST7319ea on the growth of *Listeria innocua* ATCC33090, *Listeria monocytogenes* ATCC15313, and *Ent. faecium* VRE19

(A) Bacteriocin ST651ea

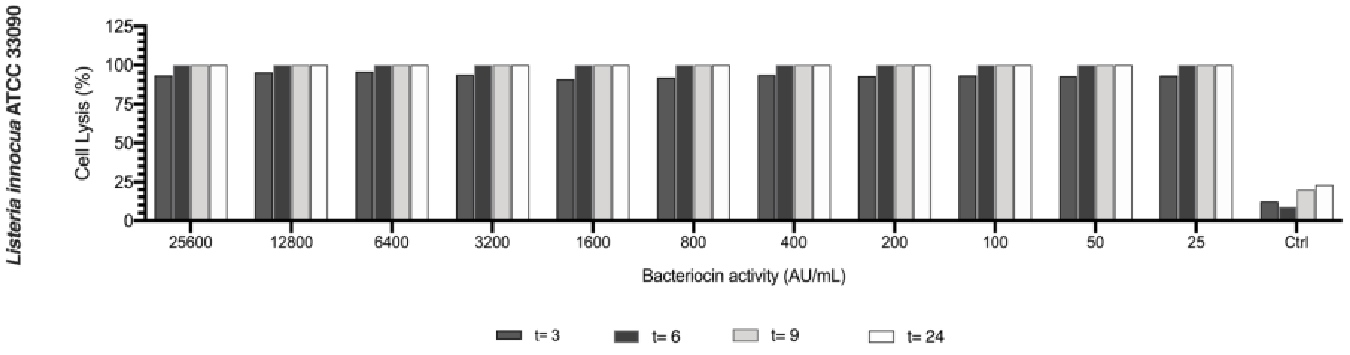

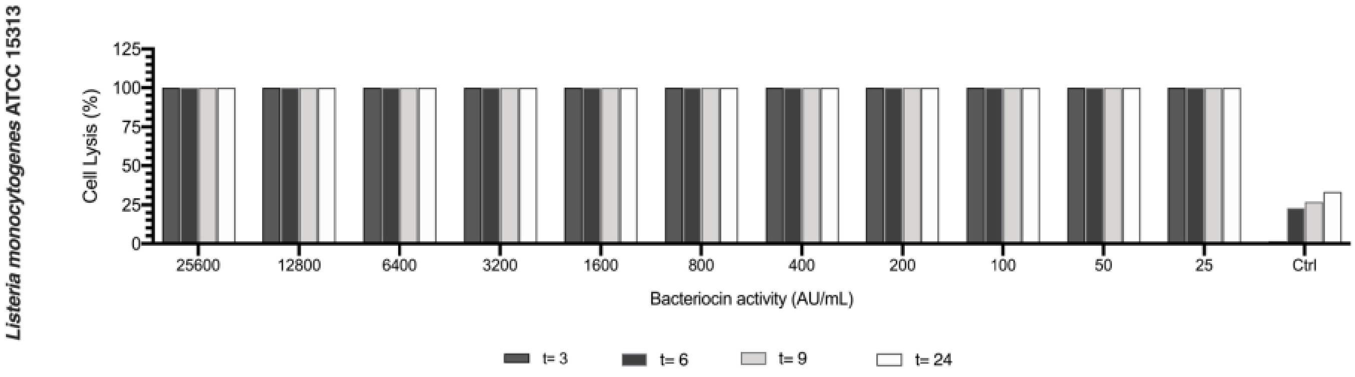

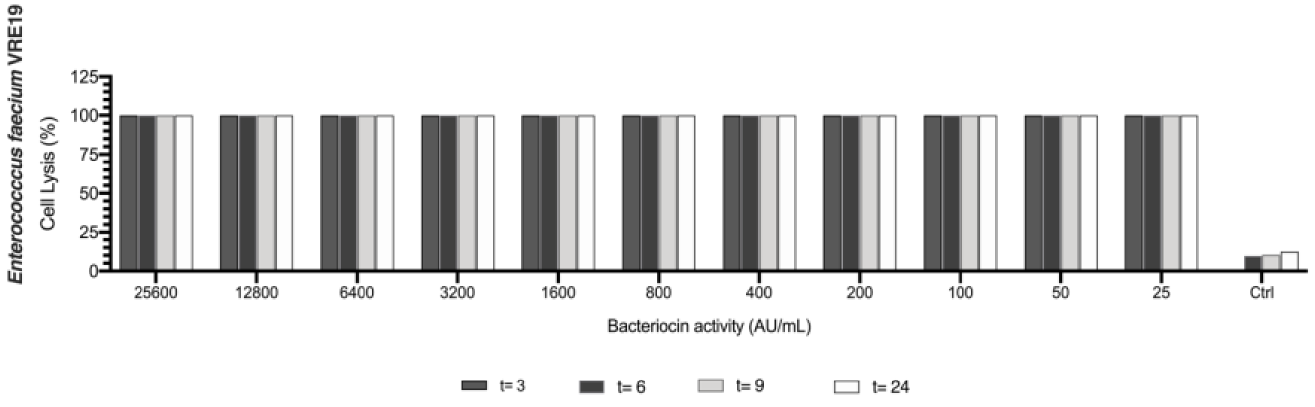

(B) Bacteriocin ST7119ea

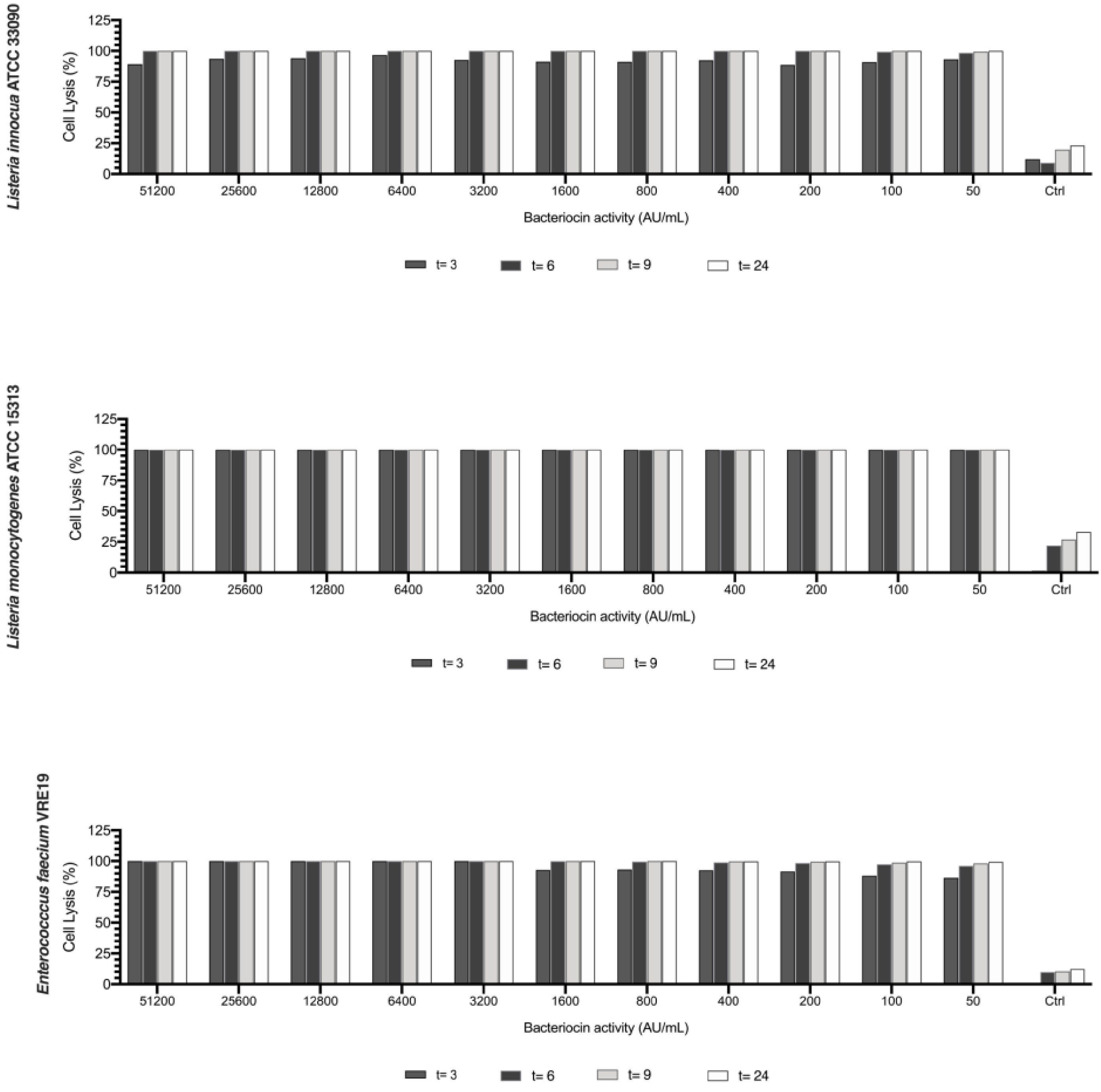

(C) **Bacteriocin ST7319ea**

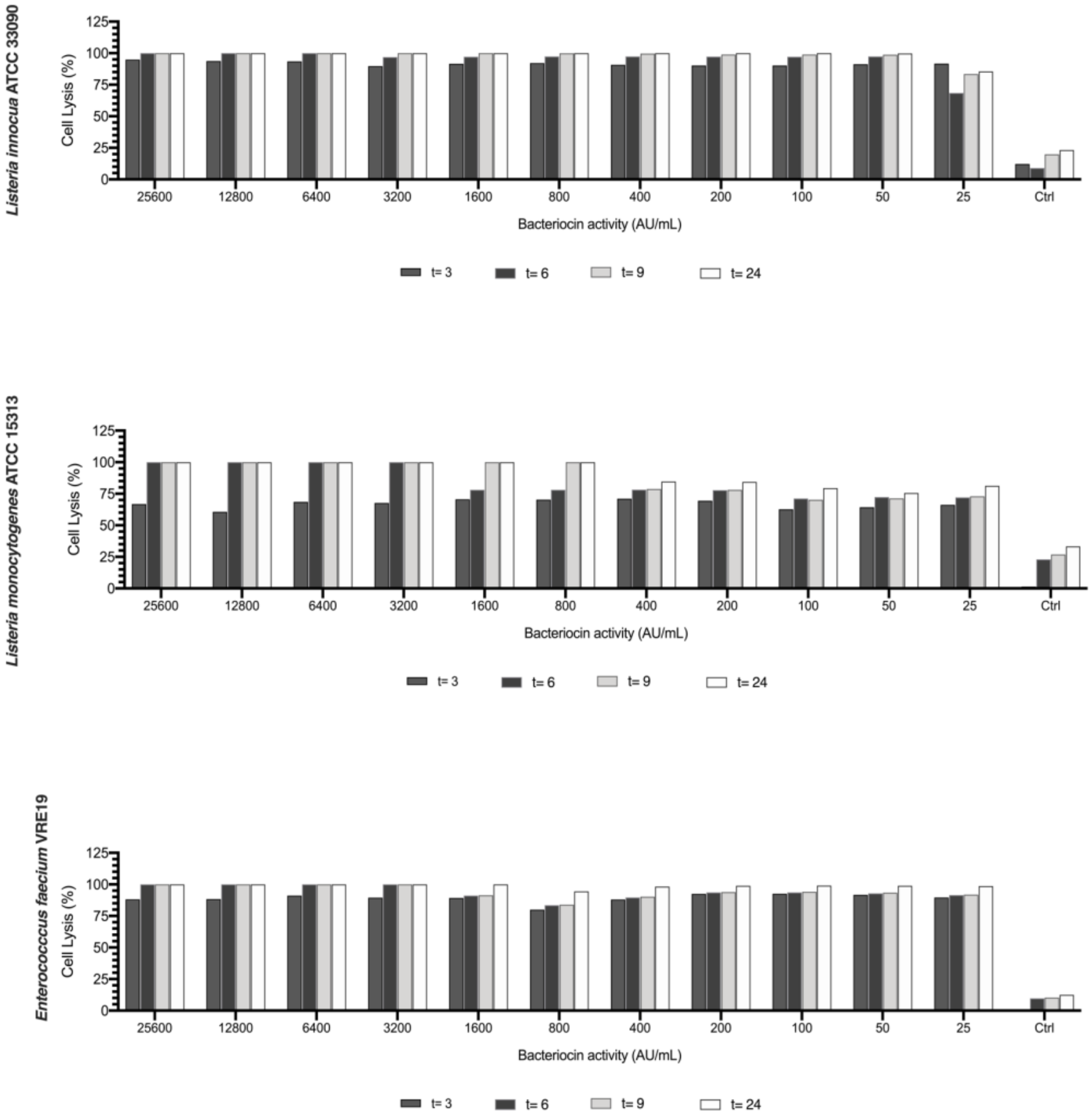

Figure S5. Rate of cell lysis (%) of non-metabolically active *Listeria innocua* ATCC 33090, *Listeria monocytogenes* ATCC 15313, and *Enterococcus faecium* VRE19 treated with various concentrations of partially purified **(A)** bacteriocin ST651ea**, (B)** bacteriocin ST7119ea and **(C)** bacteriocin ST7319ea **(**on periods 3h, 6h, 9h, and 24h.
